# Fibroblast growth factor-21 as a novel metabolic factor for regulating thrombotic homeostasis

**DOI:** 10.1038/s41598-021-00906-2

**Published:** 2022-01-10

**Authors:** Shuai Li, Haibo Jia, Zhihang Liu, Nan Wang, Xiaochen Guo, Muhua Cao, Fang Fang, Jiarui Yang, Junyan Li, Qi He, Rui Guo, Teng Zhang, Kai Kang, Zongbao Wang, Shijie Liu, Yukai Cao, Xinghao Jiang, Guiping Ren, Kai Wang, Bo Yu, Wei Xiao, Deshan Li

**Affiliations:** 1 State Key Laboratory of New-Tech for Chinese Medicine Pharmaceutical Process, Jiangsu Kanion Parmaceutical CO. LTD, Lianyungang, 222001 People’s Republic of China; 2 College of Life Sciences and Agriculture and Forestry, Qiqihar University, Qiqihar, 161006 People’s Republic of China; 3 Bio-Pharmaceutical Lab, Life Science College, Northeast Agricultural University, Harbin, 150030 People’s Republic of China; 4 Department of Cardiology, The 2nd Affiliated Hospital of Harbin Medical University, The Key Laboratory of Myocardial Ischemia, Chinese Ministry of Education, 246 Xuefu Road, Nangang District, Harbin, 150086 Heilongjiang People’s Republic of China; 5 Molecular Imaging Research Center, Harbin Medical University, TOF-PET/CT/MR Center, The Fourth Hospital of Harbin Medical University, Harbin, 150028 People’s Republic of China

**Keywords:** Drug discovery, Molecular biology, Molecular medicine

## Abstract

Fibroblast growth factor-21 (FGF-21) performs a wide range of biological functions in organisms. Here, we report for the first time that FGF-21 suppresses thrombus formation with no notable risk of bleeding. Prophylactic and therapeutic administration of FGF-21 significantly improved the degree of vascular stenosis and reduced the thrombus area, volume and burden. We determined the antithrombotic mechanism of FGF-21, demonstrating that FGF-21 exhibits an anticoagulant effect by inhibiting the expression and activity of factor VII (FVII). FGF-21 exerts an antiplatelet effect by inhibiting platelet activation. FGF-21 enhances fibrinolysis by promoting tissue plasminogen activator (tPA) expression and activation, while inhibiting plasminogen activator inhibitor 1 (PAI-1) expression and activation. We further found that FGF-21 mediated the expression and activation of tPA and PAI-1 by regulating the ERK1/2 and TGF-β/Smad2 pathways, respectively. In addition, we found that FGF-21 inhibits the expression of inflammatory factors in thrombosis by regulating the NF-κB pathway.

## Introduction

Cardiovascular disease (CVD) has become an important global public health issue, and thrombosis is a major factor leading to the development of CVDs^1^. If not treated in a timely manner, thrombosis develops into myocardial infarction, stroke and venous thromboembolism, which are life threatening. Anticoagulants prevent the activation of clotting factors and their involvement in thrombosis. These drugs are diverse and complex, and if used improperly, anticoagulants can cause serious bleeding complications, necessitating adaptation adjustment of the dose^2^. Thrombolytic agents, which are provided by intravenous administration of thrombolytic drugs, have a bleeding risk^3^. Therefore, identifying appropriate medicines for thrombosis has become a goal of researchers worldwide.

FGF-21 is a member of the fibroblast growth factor (FGF) family and preferentially signals to tissues through a receptor complex consisting of FGFR and β-klotho. β-klotho is essential for FGF-21’s interaction with FGFR to activate their downstream signaling pathways^4,5^. FGF-21 has multiple appreciated roles in physiology including potent effects on obesity, clearance of systemic glucose and lipids, improvement of insulin sensitivity and inhibition of oxidative stress^6–8^. Thus, FGF-21 is believed to be associated with the most prevalent human chronic diseases such as obesity, diabetes and coronary artery diseases^9^. Thrombosis is a major complication of these diseases. C-reactive protein (CRP), interleukin 6 (IL-6) and fibrinogen are associated with obesity and affect vascular structure and endothelial function^10^. In addition, hyperglycemia accelerates the formation of advanced glycation end products, which cause endothelial dysfunction. Hyperglycemic oxidative stress has been reported to promote the formation of thrombin and platelet activation in diabetic patients^11^. Of note, multiple studies have demonstrated that FGF-21 exerts pleiotropic effects in regulating glycolipid metabolism, inflammatory reactions and other effects. Peroxisome proliferator-activated receptor γ (PPARγ), an agonist of FGF-21, inhibits platelet activation and intra-arterial thrombus formation by increasing the expression of constitutive nitric oxide synthase (cNOS) and thrombomodulin^12^. A recent study reported that PPARγ suppresses angiotensin matrix proteins, such as collagen, fibronectin and laminin^13^. Based on these previous findings, we hypothesized that FGF-21 may play a role in thrombosis. To test this hypothesis, we analyzed an animal model of thrombosis and found that FGF-21 suppresses thrombus formation and conveys little risk of bleeding. Furthermore, we found that FGF-21 regulates thrombosis through the following mechanisms: (1) anticoagulation by inhibiting the expression and activation of factor VII (FVII); (2) antiplatelet therapy by inhibiting platelet activation; (3) fibrinolysis by promoting tissue plasminogen activator (tPA) expression and activation; (4) attenuation of inflammation in response to thrombus.

## Materials and methods

### FGF-21 preparation

Mouse FGF21 was cloned into a commercial *Escherichia. coli* (*E. coil*) expression vector, pSUMO (LifeSeusors Inc). The recombinant plasmid was transformed into the host bacterium Rossetta (DE3). A single colony was inoculated into LB media containing ampicillin (100 μg/ml). When the OD_600_ reached 0.4–0.6, 0.25 mmol/L isopropyl-β-d-thiogalactoside (IPTG) was added to the medium. The recombinant FGF21 protein was purified using a Ni Sepharose 6 Fast Flow column in an AKTA purifier (GE Healthcare)^8^. The concentration of purified protein was measured using a BCA protein assay (BCA Protein Assay Kit, Thermo, USA) and stored at − 80 °C until use.

### Animals and thrombosis model

Animals: Male New Zealand rabbits (2.5–3 kg) and male ICR mice (20–30 g) were purchased from Changchun Yisi Animal Institute. All animal experiments were performed in strict accordance with the protocols for the Guide for the Care and Use of Laboratory Animals of the National Institutes of Health and were approved by the Harbin Veterinary Research Institute Animal Care and Use Committee. All methods in the manuscript follow the recommendations in the ARRIVE guidelines.

The arterial thrombosis model was induced using FeCl_3_ as previously described^14^. Briefly, in the prevention experiment, rabbits were treated with or without FGF-21 (10 mg/kg) via intravenous injection 5 days prior to FeCl_3_ treatment. Rabbits were anesthetized using chloral hydrate (10%, 1.2 ml/kg) via intravenous injection. Filter paper (1 × 2 mm) soaked in 20% FeCl_3_ was wrapped around the external surface of the carotid artery for 10 min. Imaging of the thrombus was detected using OCT and DSA^15^. OCT and DSA tests were detected immediately after FeCl_3_ was applied to the carotid artery for 10 min. In the treatment experiment, rabbits were treated with or without FGF-21 (10 mg/kg) immediately after FeCl_3_-induced thrombus formation as mentioned previously. OCT and DSA detection was performed 2 h later.

The thrombosis mouse model was induced using carrageenan (Ca) in combination with lipopolysaccharide (LPS) as previously described^16^. Ca (3 mg/kg, Sigma Chemical Co) was administered via intraperitoneal injection in mice. After 16 h, LPS from *Escherichia coli* serotype 026:B6 (Sigma-Aldrich, St Louis, MO) was administered via the tail vein at a dose of 50 μg/kg. Then, FGF-21 (FL: 5 mg/kg; FH: 10 mg/kg) or vehicle was administered to the mice once daily for 5 days.

### OCT and DSA imaging and analysis

Optical coherence tomography (OCT) imaging and analysis were performed as previously described^15^. In brief, an intravascular OCT imaging catheter (ImageWire, M2, LightLab Imaging, Westford, Massachusetts) was inserted through the cut down section of the carotid artery. Serial OCT images were obtained in an automated pullback format at a rate of 3 mm/s. OCT images were analyzed using the offline software program provided by LightLab Imaging Inc. In each OCT frame, the lumen area (LA) and flow area (FA) were measured, and the thrombus area (TA), thrombus volume (TV) and thrombus burden (TB) were calculated as follows:

$$\begin{aligned} {\text{TA }}\left( {{\text{mm}}^{{2}} } \right) & = {\text{ LA }}\left( {{\text{mm}}^{{2}} } \right){-}{\text{FA }}\left( {{\text{mm}}^{{2}} } \right); \\ {\text{TV }}\left( {{\text{mm}}^{{3}} } \right) & = {\text{ mean TA }}\left( {{\text{mm}}^{{2}} } \right) \times {\text{Thrombus length }}\left( {{\text{mm}}} \right); \\ {\text{TB }}\left( \% \right) & = {\text{ mean TA }}\left( {{\text{mm}}^{{2}} } \right)/{\text{mean LA }}\left( {{\text{mm}}^{{2}} } \right) \times {1}00\% . \\ \end{aligned}$$

Using the digital subtraction angiography (DSA) of TOSHIBA infinix-1, the femoral artery of the rabbit was punctured using an 18 G needle, a 4 F catheter sheath was inserted into the exchange guidewire, a 1.8 F microcatheter was introduced, the catheter head was placed in the aortic arch for angiography, and imaging results were obtained by injection of contrast fluid.

### Tail bleeding time

Tail bleeding was performed as previously described^17^. In brief, mice were placed in a prone position and the distal 3 mm section of the tail was removed using a scalpel. The tail was then immediately soaked in isosmotic salt water at 37 °C. The bleeding time was subsequently observed and recorded.

### Platelet preparation

Platelets were prepared using a platelet separation kit (P1620, Solarbio) according to the manufacturer`s instructions. Fresh anticoagulated whole blood (EDTA) was collected and diluted with an equal volume of tissue diluent. The separation solution was then added to the centrifuge tube at 2× the blood volume. The diluted blood was then spread on top of the separation liquid level, and the interface between the two liquid levels was kept clear. Centrifugation was performed at 250 g at room temperature for 15 min. The platelet-rich plasma layer was then carefully absorbed into a 15 mL clean centrifuge tube, 10 mL cell washing solution was added, and the sample was centrifuged at 500 g for 20 min. The supernatant was discarded, and the platelets were resuspended in 5 mL cell washing solution, and centrifuged at 500 g for 20 min. The supernatant was then discarded, and the pellet was suspended again.

### Cell culture and treatments

EA.hy926 (human umbilical vein cell fusion cell) cells were purchased from the American Type Culture Collection (ATCC). EA.hy926 cells were cultured in Dulbecco’s modified Eagle’s medium (DMEM, Gibco, USA) containing 10% FBS and 1%antibiotics (penicillin–streptomycin) in a CO_2_ incubator (5% CO_2_) at 37 °C. The cells were subsequently starved for 12 h in serum-free medium before the experiment. Cells were divided into the control, LPS (LPS 1000 μg/mL), FL (LPS 1000 μg/mL + FGF-21 0.1 M), and FH (LPS 1000 μg/mL + FGF-21 1.0 M) groups. After treatment, cells in each group were cultured for an additional 12 h.

### Preparation and transfection of siRNA

Homo β-klotho-siRNA was synthesized by Sangon Biotech (Shanghai). The sense and antisense strands of β-klotho-siRNA were as follows: CAUCCACACACACCUUAAATT (sense) and UUUAAGGUGUGUGUGGAAUGTT (antisense). The sense and antisense strands of NC (non-targeting siRNA) were as follows: UUCUCCGAACGUGUCACGUTT (sense) and ACGUGACACGUUCGGAGAATT (antisense). Cultured EA.hy926 cells were transfected with siRNA using Lipofectamine 3000 transfection reagent (Invitrogen).

### Enzyme-linked immunosorbent assays (ELISA)

The concentrations of FVII (EM1041, FineTest), sP-selectin (HD32526, DIBCO), CRP (MCRP00, R&D), TNF-α (MTA00B, R&D), IL-6 (M6000B, R&D), D-dimer (CSBE13584m, CUSABIO), tPA (SEA525Mu, Cloud clone Corp) and PAI-1 (SEA532Mu, Cloud clone Corp) were measured using ELISA kits according to the manufacturer`s instructions. The activity of, tPA (PA90, Oxford) and PAI-1 (IMSPAI1KTA, Innovative Research) in mouse serum was also measured using ELISA kits. For the detection of other indicators, after 5 days of treatment, blood was collected from the in each group mice via the venous sinus and was tested by ELISA.

### Real-time PCR

Total RNA was isolated using TRIzol reagent (Invitrogen, USA) according to the manufacturer’s instructions. Five micrograms of total RNA were reverse transcribed into cDNA using an MMLV first stand cDNA synthesis kit (Promega, USA)^8^. The cDNA was submitted for real-time quantitative PCR using SYBR green (ABI 7500, Applied Biosystems, Carlsbad, CA, USA)^8^.

### Western blot analysis

Vascular tissues and cells were prepared according to immunoprecipitation assay (RIPA) buffer (Beyotime Institute of Biotechnology) instructions. Protein concentrations were measured using the Pierce BCA Protein Assay Kit (Thermo, USA). We used 30–40 μg protein for sodium dodecyl sulfate polyacrylamide gel electrophoresis (SDS-PAGE), and then transferred the proteins to nitrocellulose membranes (GE Healthcare, USA)^8^. We then blocked the membranes with 5% skin milk in phosphate-buffered saline (PBS) and probed them with the following primary antibodies overnight at 4 °C: ERK1/2 (ab17942, Abcam), pERK1/2 (9102S, CST), TGF-β (ab179695, Abcam), Smad2 (ab33875, Abcam), pSmad2 (ab188334, Abcam), NF-κB p65 (ab16502, Abcam), IκBα (4812S, CST), pIκBα (2859S, CST), β actin (4970S, CST), and Lamin b1 (13435S, CST), followed by horseradish peroxidase-conjugated secondary antibodies to rabbit IgG for 1 h at 37 °C. Blots were visualized using an enhanced chemiluminescence kit (ECL; Thermo Scientific, USA)^8^. All original blots are shown in the supplementary material, but the original blots in Fig. 4d for Lamin b1, Fig. 6 for Smad 2, S2A and S4B were not saved, and only the images cropped after scanning were saved.

### Statistical analysis

Cell culture experiments were repeated three times. All analyses were performed using GraphPad Prism 5. Data were analyzed using one-way analysis of variance (ANOVA) or two-way ANOVA with post hoc comparisons performed using the Student-Newman–Keuls test. Data are expressed as the mean ± standard deviation. Statistical analysis was conducted using SPSS software and was considered statistically significant when *p* < 0.05.

## Results

### FGF-21 ameliorates FeCl_3_-induced carotid arterial thrombosis

**Figure 1:**
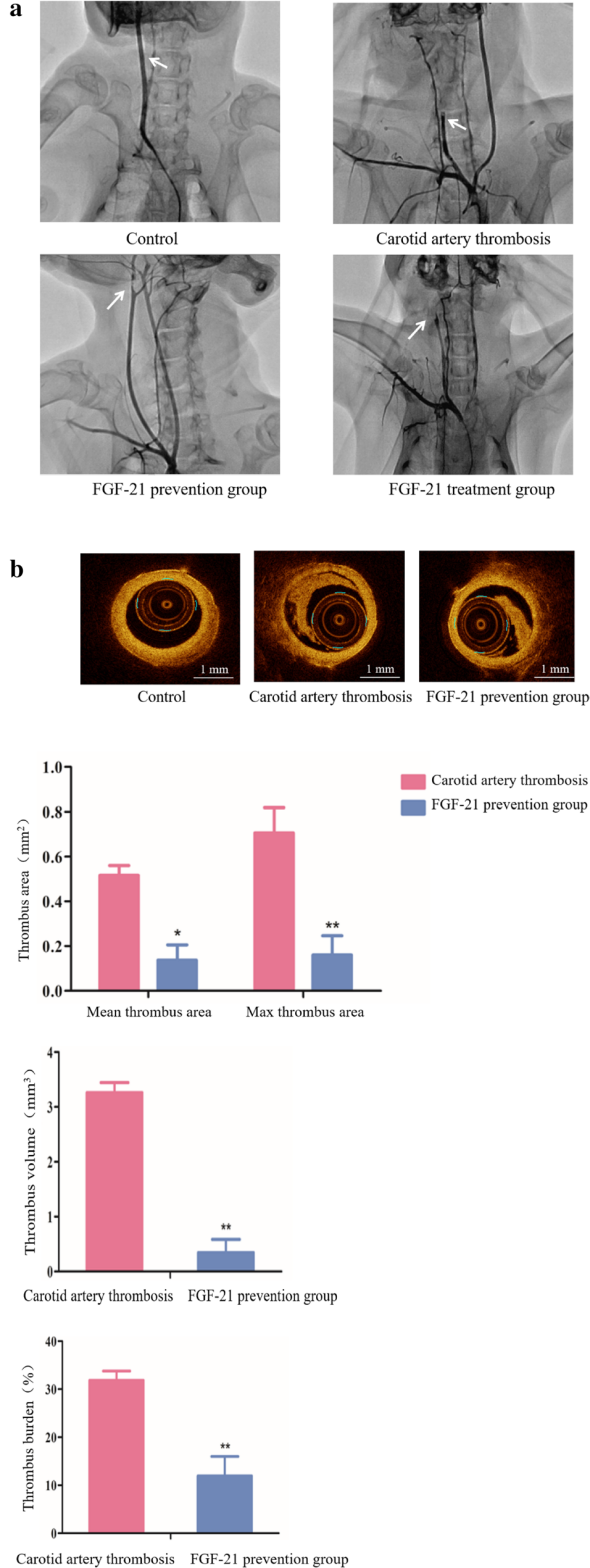

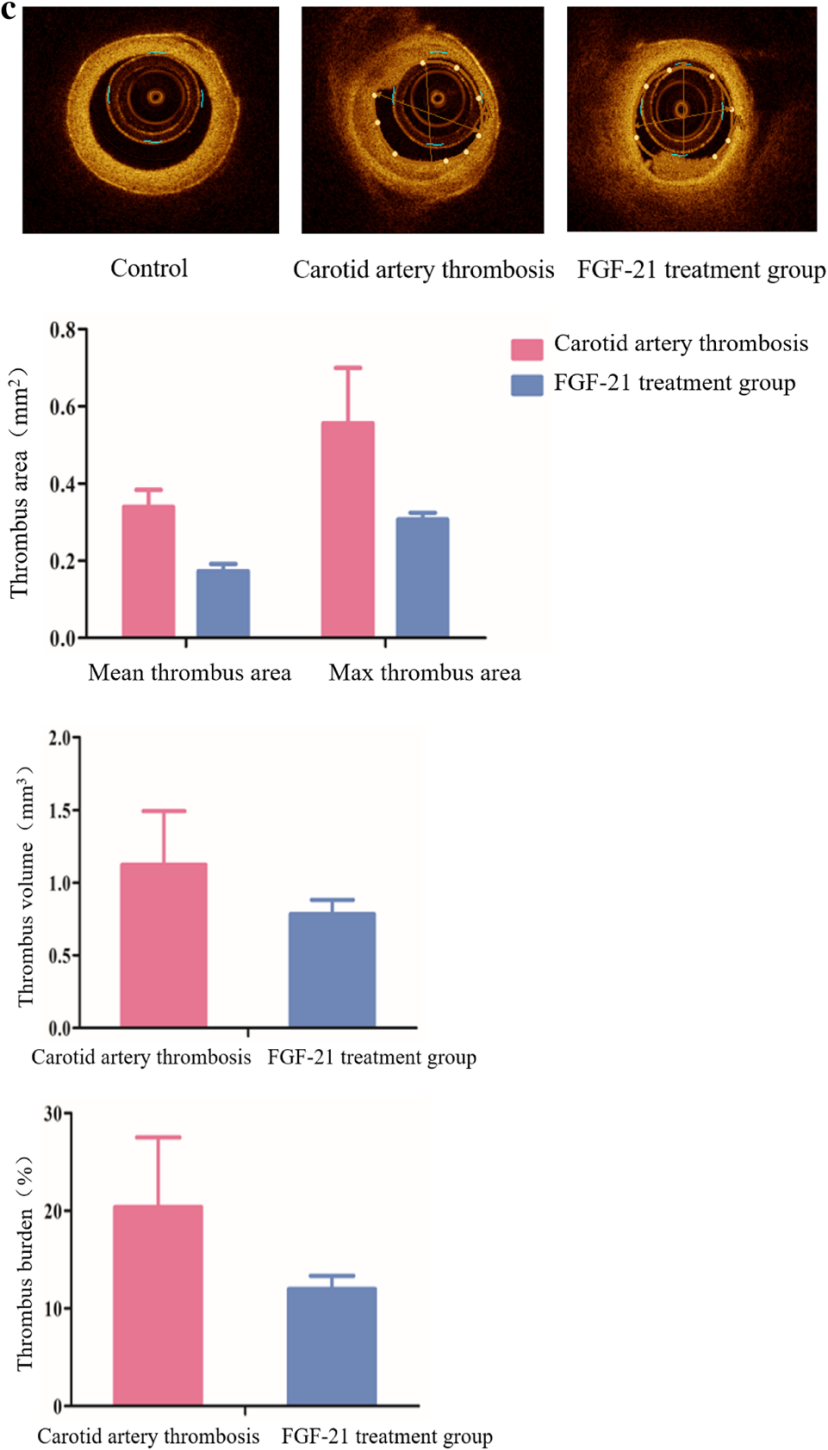
FGF-21 contributes to ameliorating FeCl_3_-induced carotid artery thrombosis in rabbits. (**a**) Representative digital subtraction angiography (DSA) images of the carotid artery. Rabbits in the FGF-21 prevention group were treated with FGF-21 (10 mg/kg) 5 days prior to FeCl_3_ treatment. Rabbits were treated with FGF-21 (10 mg/kg) for 2 h after FeCl_3_-induced carotid thrombosis formation in the FGF-21 treatment group.Control rabbits were not injured. Imaging of the thrombi was performed using DSA. The white arrow indicates the narrow position. (**b**-**c**) Representative optical coherence tomography (OCT) images of the carotid artery. Rabbits in the FGF-21 prevention group were treated with FGF-21 (10 mg/kg) 5 days prior to FeCl_3_ treatment. Rabbits were treated with FGF-21 (10 mg/kg) for 2 h after FeCl_3_-induced thrombosis formation in the FGF-21 treatment group. Control rabbits were not injured. Imaging of the thrombi was performed using OCT. The white arrow indicates the location of the white thrombus. Analysis of the mean thrombus area, maximum thrombus area, thrombus volume and thrombus burden in rabbits of each group. For all the bar graphs, data are expressed as the mean±SD (n = 6). One-way ANOVA was used for multiple group comparisons, followed by Student’s two-tailed *t* test. *p < 0.05, **p < 0.01 compare with carotid artery thrombosis.

FeCl_3_-induced vascular injury is a widely used model of occlusive thrombosis. This model is based on redox-induced endothelial cell injury, which is simple and sensitive to both anticoagulation and thrombolytic drugs^18^. FeCl_3_ (20%) was used to treat the carotid artery of New Zealand rabbits for 10 min. Using DSA detection, we observed that the right carotid artery of the FeCl_3_-induced rabbits was completely occluded due to thrombosis, so that the distal end could not be developed. The right carotid artery of FGF-21 prevention rabbits and treatment rabbits displayed severe local stenosis in the vascular lumen and irregular filling defects in the blood vessels, but these symptoms were attenuated compared with the FeCl_3_-induced rabbits (Fig. 1a). These results suggest that FGF-21 prevents and attenuates thrombosis. To further characterize the antithrombotic effect of FGF-21, OCT was used to assess the lesion site. OCT is a high-resolution intracoronary imaging modality that has been shown to be able to differentiate plaque erosion from plaque rupture in vivo^15^. The carotid artery lumen of the control rabbits was smooth without thrombosis. In the prevention trial, a large amount of white thrombus formed in the rabbits’ carotid arteries that was induced by FeCl_3_. The average thrombus area was 0.52 mm^2^, the maximum thrombus area was 0.73 mm^2^, the thrombus volume was 3.26 mm^3^ and the thrombus burden was 31.85%. A smaller amount of white thrombus formed in the carotid artery lumen of FGF-21 treated rabbits (the white thrombus exhibited reduced surface reflection, uniform signal and low attenuation). The average thrombus area was 0.14 mm^2^, the maximum thrombus area was 0.16 mm^2^, the thrombus volume was 0.35 mm^3^ and the thrombus burden was 11.93%. These indices were significantly lower than those in FeCl_3_-induced rabbits that were not treated with FGF-21 (*P < 0.05, **P < 0.01) (Fig. 1b). In the treatment trial, large amounts of white thrombi formed in the carotid lumen of FeCl_3_-induced rabbits. The average thrombus area was 0.34 mm^2^, the maximum thrombus area was 0.56 mm^2^, the thrombus volume was 1.12 mm^3^ and the thrombus burden was 20.39%. A smaller amount of white thrombus was formed in the carotid artery lumen of FGF-21 treated rabbits. The average thrombus area was 0.17 mm^2^, the maximum thrombus area was 0.31 mm^2^, the thrombus volume was 0.78 mm^3^ and the thrombus burden was 12.01%. These indices were lower than those in the FeCl_3_-induced rabbits that were not treated with FGF-21 (Fig. 1c). These results indicate that FGF-21 prevents and attenuates carotid artery thrombosis induced by FeCl_3_ in rabbits.

### FGF-21 ameliorate Ca and LPS-induced thrombosis

**Figure 2:**
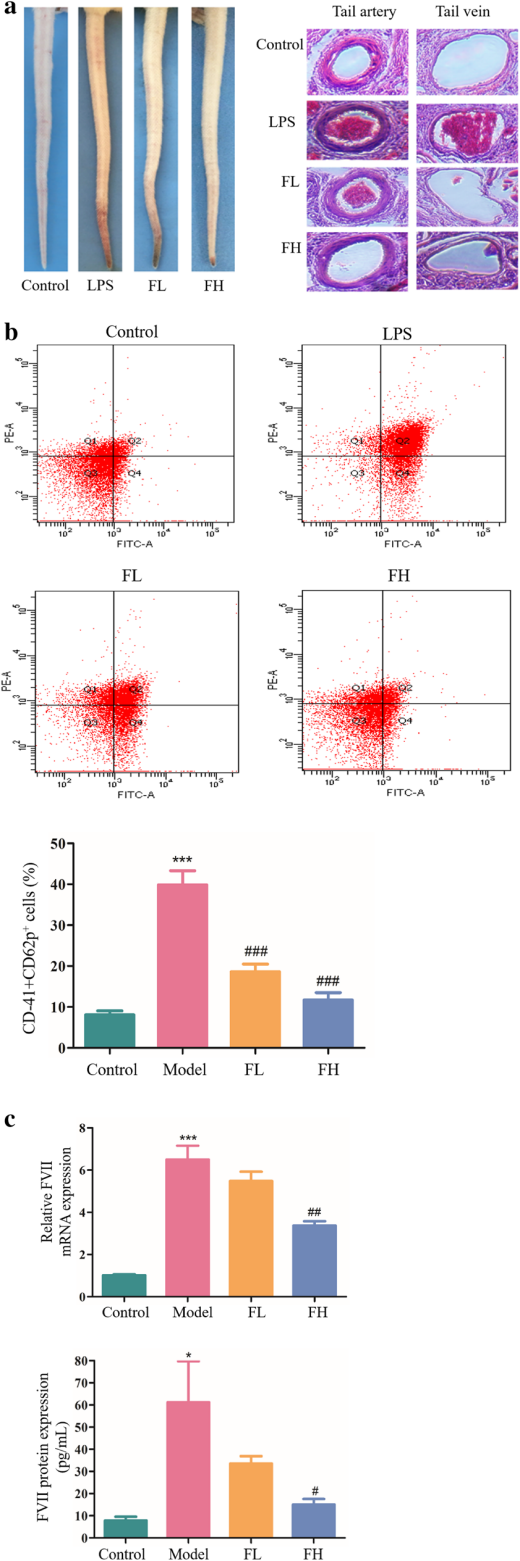
FGF-21 contributes to ameliorating Ca- and LPS-induced tail thrombosis in mice. Mice were administered Ca (3 mg/kg) via intraperitoneal injection and LPS (50 µg/kg) via the tail vein 16 h later. Then, FGF-21 was administered to the mice once daily for 5 days. Control: normal control; Model: Ca-LPS-induced thrombosis mice; FL: thrombosis mice treated with low-dose FGF-21 (5 mg/kg); FH: thrombosis mice treated with high-dose FGF-21 (10 mg/kg); FVII: factor VII; FVIIa: activity of FVII. (**a**) Representative image of tail and HE staining of tail artery and vein sections (×10) in mice. (**b**) CD 41+ and CD 62p+platelets from mice in each group. (**c**) Analysis of FVII expression in the liver by real-time PCR and in the serum by ELISA. For all the bar graphs, data are expressed as the mean±SD (n = 6). One-way ANOVA was used for multiple group comparisons, followed by Student’s two-tailed *t* test. *p < 0.05, **p < 0.01, ***p < 0.001 compare with Control; ^##^*P* < 0.01, ^###^*P* < 0.001 compare with Model.

To further characterize the antithrombotic effect of FGF-21, a mouse thrombosis model was established by combining administration of Ca and LPS. A disseminated intravascular coagulation (DIC) model has been established that focuses on interactions among platelets, inflammatory cytokines, and coagulation factors. Several studies have shown that LPS induces DIC^16^. Ca is an endotoxin that has the same coagulation mechanism as LPS, causing inflammation in the intima, which damages the integrity of endothelial cells. The combination of LPS and Ca accelerates the formation of thrombi. The extent of tissue ischemia was apparent at the distal end of the mouse tail after thrombosis. The skin tissue supplied by the embolized blood vessel became dark red due to tissue ischemia and hypoxia, which was markedly different from the normal skin tissue. After administration of FGF-21, the extent of ischemia was significantly attenuated, and FH treated mice were similar to normal mice. Pathological sections revealed that large amounts of thrombi were present in the tail artery of model mice, while the levels of thrombi were decreased in the low-dose FGF-21 treated mice, and the mice treated with high-dose FGF-21 were similar to normal mice. Compared with normal mice, large amounts of thrombi formed in the tail vein of model mice. In contrast, the amounts of thrombi in the tail vein of mice treated with FGF-21 were significantly reduced, and the high dose group appeared similar to normal mice (Fig. 2a). The above results suggest that FGF-21 attenuates the effect of Ca and LPS induced tail thrombosis in mice.

### FGF-21 inhibits coagulation in mice

The physiological processes related to thrombosis primarily include the coagulation, platelet and fibrinolysis systems. Platelets contain a variety of substances associated with coagulation, and abnormal platelet activity leads to thrombosis. CD 41 is a membrane glycoprotein found on the surface of platelets, and CD 62p is expressed on the surface of activated platelet membranes. The fluorescence intensity of CD 41 and CD 62p directly reflects the degree of platelet activation. Blood samples were taken from normal mice, model mice and FGF-21 treated mice. Platelet activation was clearly induced by ADP (5 μmol/L) as shown by staining with FITC-labeled CD 41 antibody and PE-labeled CD 62p antibody. The results revealed that the percentage of double stained of CD41 and CD62p platelets in normal mice (without ADP stimulation) was 6.5%, but the percentage of double stained CD41 and CD62p platelets in thrombotic mice was 45.7%, which was significantly higher than that in normal mice (***P < 0.001). The percentages of double stained CD41 and CD62p platelets in low-dose FGF-21 treated mice and high-dose FGF-21 treated mice were 21.9% and 8.3%, respectively, which were significantly lower than those in the model mice (^###^*P* < 0.001) (Fig. 2b). In addition, we assessed sP-selectin, which is considered an effective marker of platelet activation (Supplementary Fig. 1). The results showed that FGF-21 inhibited the expression of sP-selectin compared to that in thrombotic mice without FGF-21 treatment. Although the inhibitory effect of FGF21 was not statistically significant, which may be due to the activation of platelets in the process of blood collection or the detection sensitivity not being high, a dose-dependent decrease was still observed. These results indicate that FGF-21 inhibits platelet activation in the hypercoagulable state. Activation of the extrinsic coagulation pathway occurs in response to endothelial injury when tissue factor binds FVII or activated VII^19^. Once formed, the TF-VIIa complex activates both factors IX and X and initiates the coagulation process. Recent studies have identified FVIIa as a new biomarker for activated coagulation^19^. In this study, we found that expression of FVII significantly increased in thrombotic mice compared with normal mice, while FGF-21 significantly inhibited the expression of FVII compared with thrombotic mice not treated with FGF-21, and the effect in high-dose FGF-21 treated mice was significant (Fig. 2c). These results suggest that FGF-21 exerts antithrombotic effects by inhibiting platelet and FVII expression.

### FGF-21 promotes fibrinolysis with no bleeding risk

**Figure 3:**
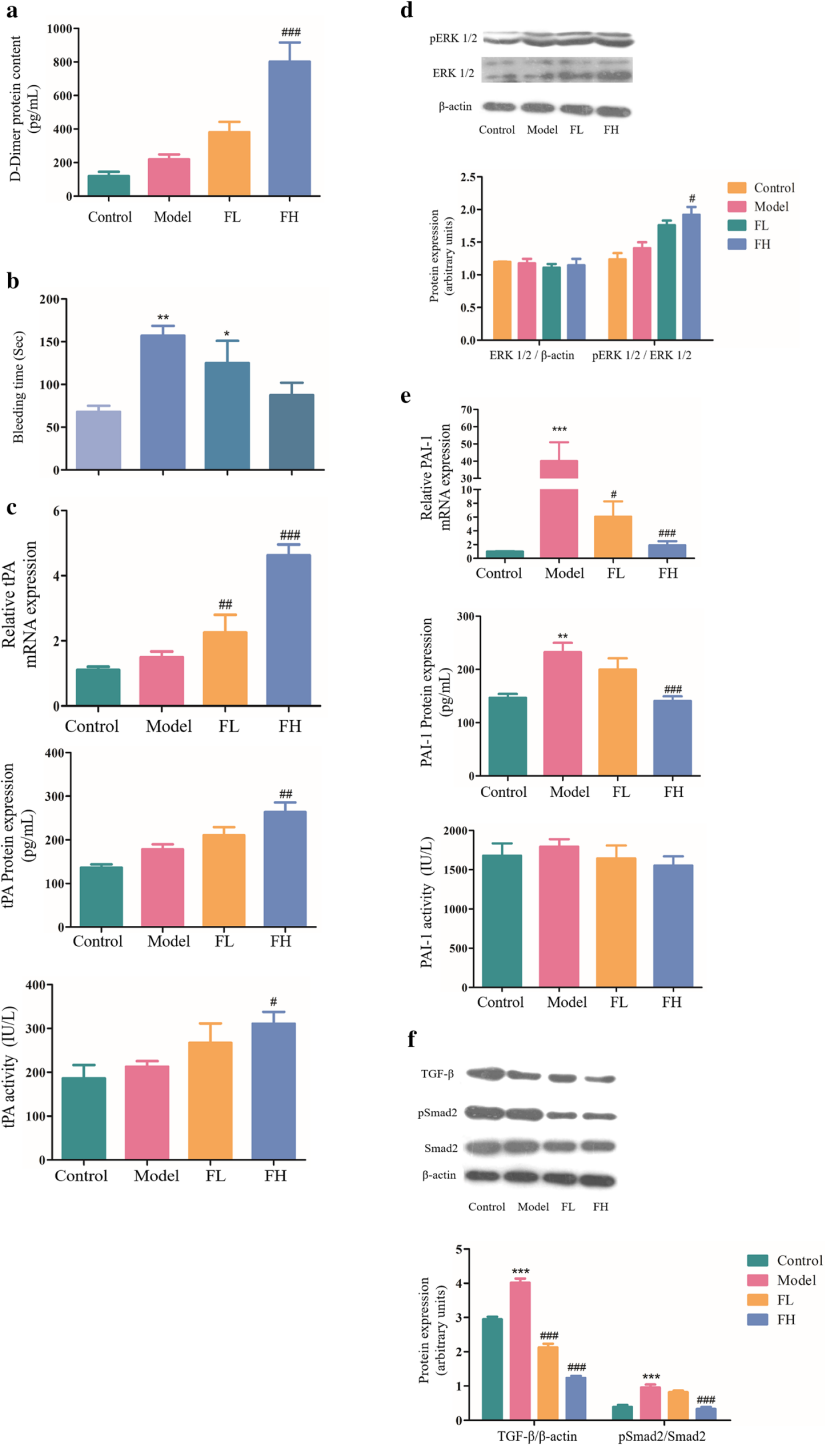
FGF-21 promotes fibrinolysis with no notable bleeding time in thrombotic mice. Mice were administered Ca (3 mg/kg) via intraperitoneal injection and LPS (50 µg/kg) via the tail vein 16 h later. Then, FGF-21 was administered to the mice once daily for 5 days. Control: normal control; Model: Ca-LPS-induced thrombosis mice; FL: thrombosis mice treated with low-dose FGF-21 (5 mg/kg); FH: thrombosis mice treated with high-dose FGF-21 (10 mg/kg); tPA: tissue plasminogen activator; PAI-1: plasminogen activator inhibitor 1. (**a**) ELISA of D-dimer production in the serum. (**b**) Bleeding time in mice of each group. Mice were treated with aspirin (2 mg/kg), warfarin (1 mg/kg) or FGF-21(20 mg/kg) for 3 days. Tail bleeding time was assessed in mice by cutting 1 cm of tail tissue from the tip. (**c**) Analysis of tPA expression by real-time PCR and ELISA in mice. The activity of tPA was analyzed by ELISA. (**d**) Western blot analysis of ERK1/2 phosphorylation levels in mice. (**e**) Analysis of PAI-1 expression by real-time PCR and ELISA in mice. The activity of PAI-1 was analyzed by ELISA. (**f**) Western blot analysis of Smad2 phosphorylation levels and TGF-β expression in mice. For all bar graphs, data are expressed as the mean±SD (n = 6). One-way ANOVA was used for multiple group comparisons, followed by Student’s two-tailed *t* test. *p < 0.05, **p < 0.01, ***p < 0.001 compared with the control; ^#^*P* < 0.05, ^##^*P* < 0.01, ^###^*P* < 0.001 compared with the model.

D-dimer has been identified as a marker of dynamic changes in fibrinolysis^19^. We found that plasma concentration of D-dimer in FGF-21 treated mice were significantly higher than that in thrombotic mice (^###^*P* < 0.001) (Fig. 3a). At present, most thrombolytic agents have the side effect of hemorrhage. To explore whether FGF-21 treatment conveys a risk of bleeding, we used a tail bleeding assay to examine hemorrhage responses after FGF-21 administration. FGF-21 was injected, and the tail bleeding time was measured for each mouse 180 min later. We found that the average tail bleeding time for normal mice was approximately 68 s. However, the bleeding time of aspirin (2 mg/kg, 157 s) and warfarin (1 mg/kg, 125 s) treated mice was significantly prolonged. The bleeding time of FGF-21 treated mice at a dose of 20 mg/kg was 88 s, which was twofold higher than the experimental dose, and there were no significant differences compared with normal mice (Fig. 3b). These results indicate that FGF-21 conveys little risk of bleeding. tPA is well known for its role in the blood coagulation system^20^. tPA thrombolytic activity is neutralized by the inhibitor PAI-1^21^. The above results indicated that FGF-21 promoted thrombolysis, and we hypothesized that FGF-21 treatment promotes thrombolysis by upregulating the expression of tPA in vivo. To test this hypothesis, the mRNA and protein levels of tPA in each mouse were detected using real-time PCR and ELISA, respectively.The results revealed that the expression of tPA in thrombotic mice was upregulated compared to that in normal mice. Treatment with FGF21 significantly increased the expression of tPA compared to that in thrombotic mice. Importantly, by ELISA, we found that the activity of tPA was increased, consistent with its expression (Fig. 3c). Previous studies have shown that the ERK1/2 signaling pathway plays a key role in regulating the expression of tPA.^22–25^ Therefore, we analyzed ERK1/2 phosphorylation in mice treated with or without FGF-21 by western blot. As shown in Fig. 3d, treatment with FGF-21 increased levels of phospho-ERK1/2 compared with model mice, and levels of phospho-ERK1/2 were significantly increased in the high FGF-21 dose group (^#^*P* < 0.05). Furthermore, the mRNA and protein levels of PAI-1 in each mouse were detected using real-time PCR and ELISA, respectively. The results revealed that the expression of PAI-1 in thrombotic mice was significantly increased compared to that in normal mice (***P < 0.001). However, treatment with FGF-21 prevented this increase. Using ELISA, we found that administration of FGF-21 also inhibited PAI-1 activity, which was consistent with the trend observed in PAI-1 concentration (Fig. 3e). When the PAI-1 expression levels change, its activity changes in the same direction, but the change in expression level precedes the change in activity. This may be the reason that FGF significantly inhibited PAI-1 expression but did not significantly inhibit PAI-1 activity. This unexpected result may also be due to insufficient sensitivity of the assay at low PAI-1 levels. TGF-β/Smad2 plays an important role in mediating the expression of PAI-1 in humans^26^. The above results demonstrated that FGF-21 inhibited PAI-1 expression in mice. Therefore, we hypothesized that FGF-21 inhibits PAI-1 expression via inactivation of the TGF-β/Smad2 pathway. Western blot assays revealed that the expression of TGF-β in model mice was significantly increased compared to that in control mice, and administration of FGF-21 blocked this increase. Furthermore, we found that levels of phospho-Smad2 in model mice were significantly upregulated. In contrast, levels of phospho-Smad2 were significantly downregulated after FGF-21 treatment (Fig. 3f). These results suggest that FGF-21 promotes fibrinolysis with no bleeding risk in mice by mediating the expression and activation of tPA and PAI-1 through regulation of the ERK1/2 and TGF-β/Smad2 pathways, respectively.

### FGF-21 suppresses the inflammatory response in thrombosis

**Figure 4:**
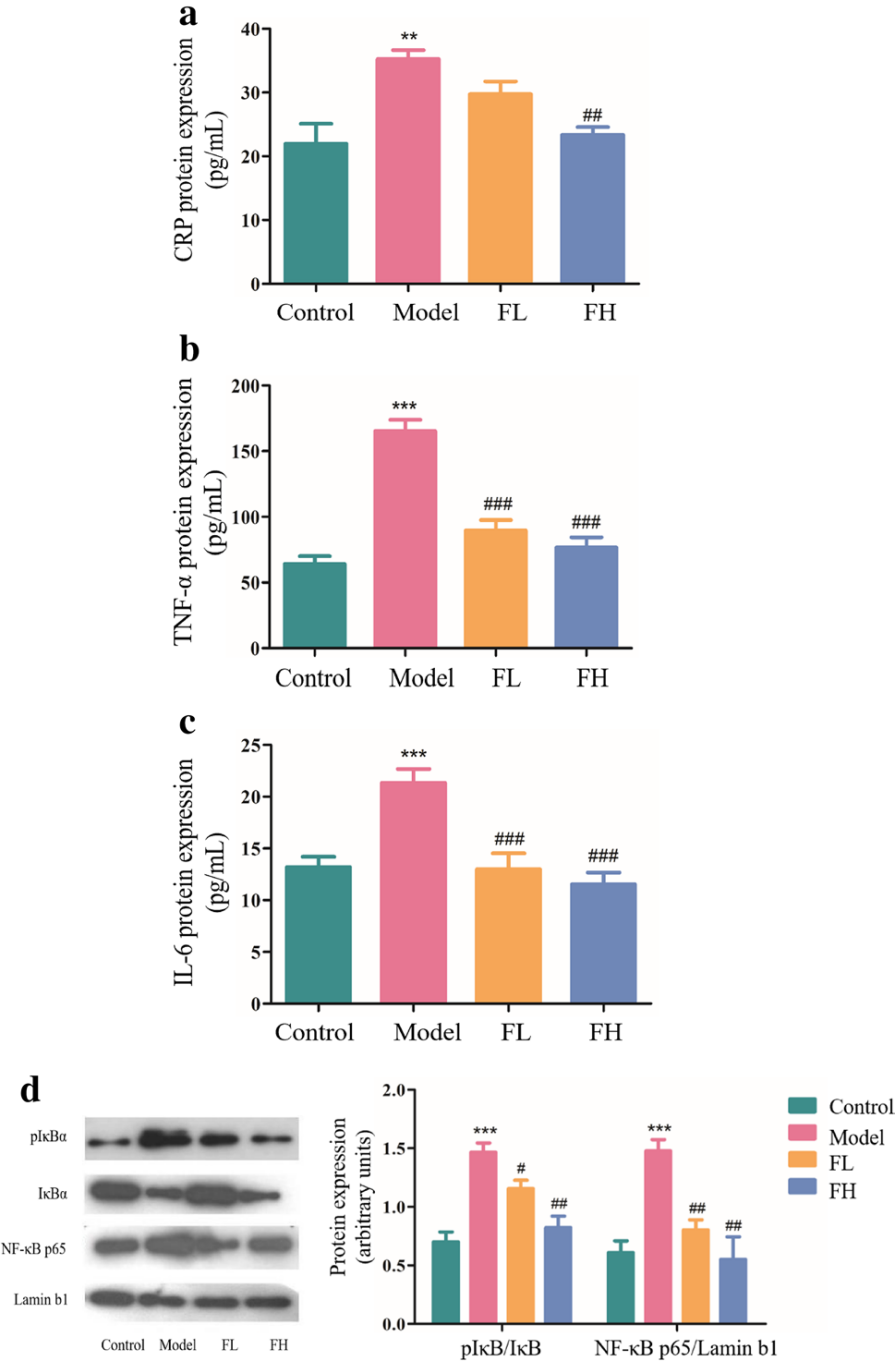
FGF-21 inhibits inflammation in thrombotic mice. Mice were administered Ca (3 mg/kg) via intraperitoneal injection and LPS (50 μg/kg) via the tail vein 16 h later. Then, FGF-21 was administered to the mice once daily for 5 days. Control: normal control; Model: Ca-LPS-induced thrombosis mice; FL: thrombosis mice treated with low dose FGF-21 (5 mg/kg); FH: thrombosis mice treated with high dose FGF-21 (10 mg/kg); CRP: c-reactive protein; TNF-α: tumor necrosis factor-α; IL-6: interleukin 6. (**a**-**c**) ELISA of CRP, TNF-α, IL-6 expression in the serum. (**d**) Western blot analysis of phospho-IκB and p65 expression in mice. For all bar graphs, data are expressed as the mean±SD (n = 6). One-way ANOVA was used for multiple group comparisons, followed by Student’s two-tailed *t* test. **p < 0.01, ***p < 0.001 compared with the control; ^#^*P* < 0.05, ^##^*P* < 0.01, ^###^*P* < 0.001 compared with the model.

The inflammatory response, coagulation and fibrinolysis system constitute a complex regulatory network, in which inflammatory factors play a very important role as both signal transducers and effector molecules.CRP is an acute phase protein synthesized by the liver, in response to inflammation, such as microbial invasion or tissue injury. Increased expression of CRP indicates activation of the inflammatory response in vivo. The results demonstrated that expression of CRP in the serum of the thrombosis mice was significantly increased compared with normal mice (**P < 0.01), while expression of CRP in FGF-21 treated mice was decreased compared with the thrombosis mice in a dose-dependent manner, and expression of CRP in high dose FGF-21 treatment mice was significantly decreased (^##^*P* < 0.01) (Fig. 4a). Tumor necrosis factor-α (TNF-α), an initiator of inflammation, induces the activation of macrophages, lymphocytes and other cells as well as the adhesion and aggregation of inflammatory cells to the vascular wall, increases blood viscosity and blood stasis, aggravates vascular endothelial injury and promotes thrombosis. The results showed that thrombotic mice exhibited significantly increased expression of TNF-α compared with normal mice (***P < 0.001). Compared to thrombotic mice, treatment with FGF-21 decreased the expression of TNF-α in a dose-dependent manner (^###^*P* < 0.001) (Fig. 4b). IL-6 stimulates the liver to produce plasminogen activator inhibitors, leading to decreased fibrinolytic function and promotion of platelet aggregation and thrombosis. The results demonstrated that thrombotic mice displayed significantly increased expression of IL-6 compared with normal mice (***P < 0.001). In contrast, we found that expression of IL-6 was significantly decreased in FGF-21 treated mice in a dose-dependent manner (^###^*P* < 0.001) (Fig. 4c). Recent studies suggest that inflammatory cytokines induce a hypercoagulable state. Inflammatory factors such as CRP, IL-6 and TNF-α have been reported to enhance the expression of PAI-1^22,23^. We hypothesized that FGF-21 ameliorates the overall inflammatory environment by inhibiting the NF-κB pathway, which is a key pathway in the inflammatory response, and consequently inhibits the coagulation state^27^. Western blot analysis revealed that levels of phospho-IκBα in thrombotic mice were significantly increased compared to that in normal mice (***P < 0.001). Administration of FGF-21 significantly inhibited levels of phospho-IκBα, and the nuclear p65 content in thrombotic mice was significantly increased compared to that in normal mice, whereas treatment with FGF-21 blocked this increase (^#^*P* < 0.05, ^##^* P* < 0.01) (Fig. 4d). These results suggest that FGF-21 mediates the coagulation state by inhibiting the NF-κB pathway, subsequently ameliorating the overall inflammatory state.

### FGF-21 inhibits the expression of inflammatory cytokines in EA.hy926 cells

**Figure 5:**
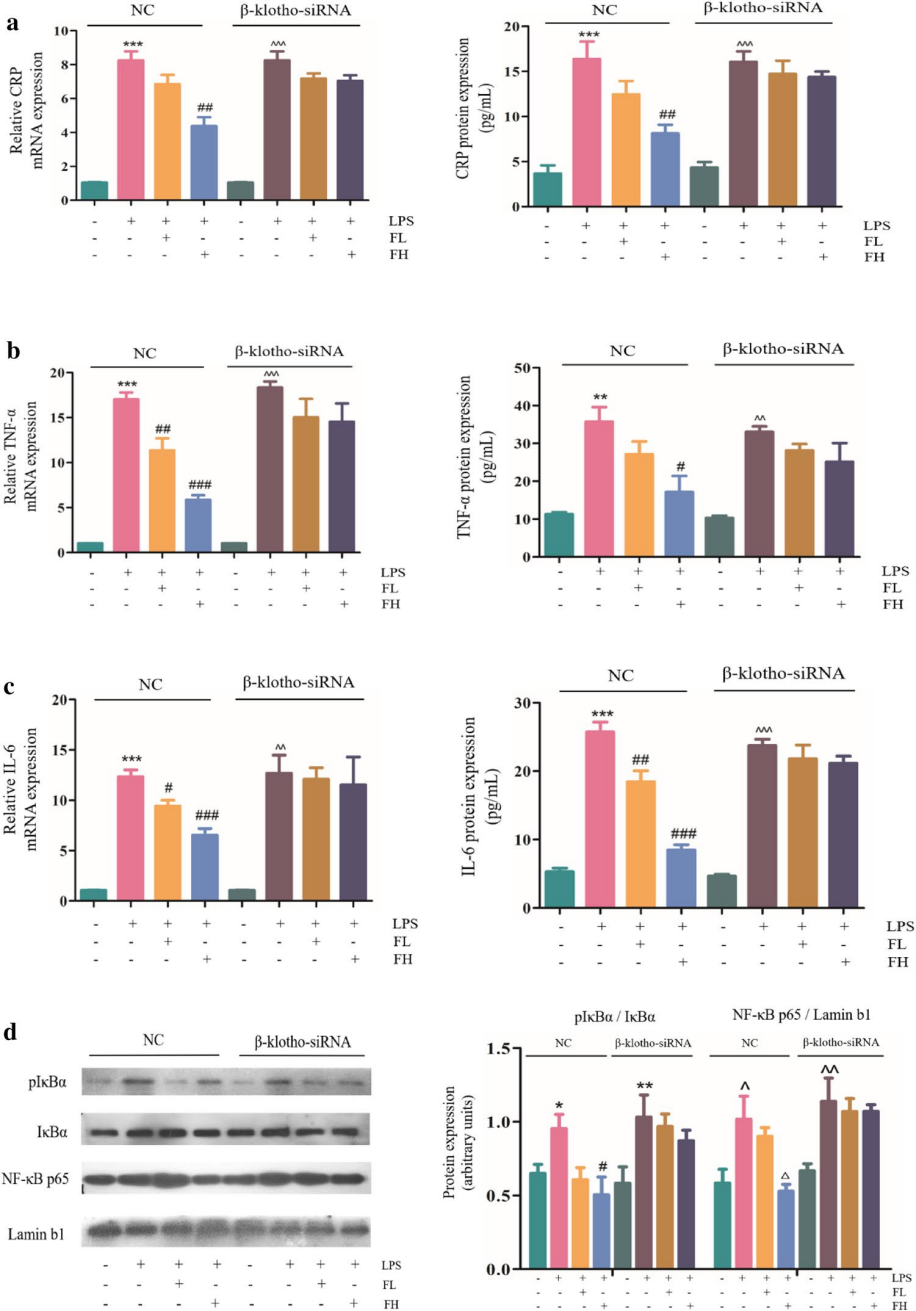
FGF-21 inhibits the expression of inflammatory cytokines in EA.hy926 cells. β-klotho-siRNA or NC siRNA was transiently transfected into EA.hy926 cells using Lipofectamine 3000 transfection reagent. Cells were treated with LPS (1000 μg/mL) with or without FGF-21 (FL: FGF-21 0.1 M, FH: FGF-21 1.0 M). CRP: C-reactive protein; TNF-α: tumor necrosis factor-α; IL-6: interleukin 6; NC: nontransfection. (**a**-**c**) Analysis of CRP, TNF-α, IL-6 expression in EA.hy926 cells by real-time PCR and ELISA. (**d**) Western blot analysis of phospho-IκB and p65 expression in EA.hy926 cells. For all bar graphs, data are expressed as the mean±SD (n = 3). One-way ANOVA was used for multiple group comparisons, followed by Student’s two-tailed *t* test. *p < 0.05, **p < 0.01, ***p < 0.001 compared with the control NC group; ^#^*P* < 0.05, ^##^*P* < 0.01, ^###^*P* < 0.001 compared with the model NC group; ^^^*P* < 0.05, ^^^^*P* < 0.01, ^^^^^*P* < 0.001 compared with the control of β-klotho-siRNA group.

FGF-21 functions primarily by binding to FGF receptors and coreceptors (β-klotho). FGF-21 cannot directly bind to FGFR when β-klotho is absent. Therefore, β-klotho is an important component mediating the biological function of FGF-21. We first determined that β-klotho is expressed in EA.hy926 cells (Supplementary Fig. 2). Then, we determined the concentration and duration of LPS (1000 μg/mL, 12 h) that induced cellular damage (Supplementary Fig. 3). EA.hy926 is a human umbilical vein cell fusion line that is widely used in vascular-related research. LPS causes inflammation in the endangium, which damages the integrity of endothelial cells. In this study, LPS was used to induce EA.hy926 cell injury, which was similar to the clinical cause of thrombosis. The experiment was divided into normal (Control), LPS-treated (Model, LPS 1000 μg/mL), LPS and low-dose FGF-21 cotreated (FL, LPS 1000 μg/mL + FGF-21 0.1 M), and LPS and high-dose FGF-21 cotreated groups (FH, LPS 1000 μg/mL + FGF-21 1.0 M). In NC group (transfection of non-targeting siRNA), compared with control cells, expression of CRP, TNF-α and IL-6 was significantly increased in LPS-treated cells (**P < 0.01, *** *P* < 0.001). Compared with LPS-treated cells, expression of CRP, TNF-α and IL-6 was significantly decreased in FGF-21 treated cells in a dose-dependent manner (^#^*P* < 0.05, ^##^*P* < 0.01, ^###^*P* < 0.001). The results demonstrated that FGF-21 inhibits phospho-IκBα and nuclear p65 induced by LPS in EA.hy926 cells, consistent with the in vivo experiments. However, FGF-21 treatment had no effect on the expression of CRP, TNF-α, IL-6, IκBα and nuclear p65 in the β-klotho-siRNA group (Fig. 5a-c). The results demonstrated that when β-klotho was present, FGF-21 reduced the expression of inflammatory factors and the accumulation of IκBα phosphorylation and nuclear p65 in EA.hy926 cells. However, after β-klotho knockdown, the expression of inflammatory factors and the accumulation of IκBα phosphorylation and nuclear p65 were not significantly reduced, suggesting that FGF-21 has an inhibitory effect on the inflammatory response that occurs through the NF-κB pathway in EA.hy926 cells and is β-klotho-dependent (Fig. 5d).

### FGF-21 promotes tPA expression and inhibits PAI-1 expression in EA.hy926 cells

**Figure 6:**
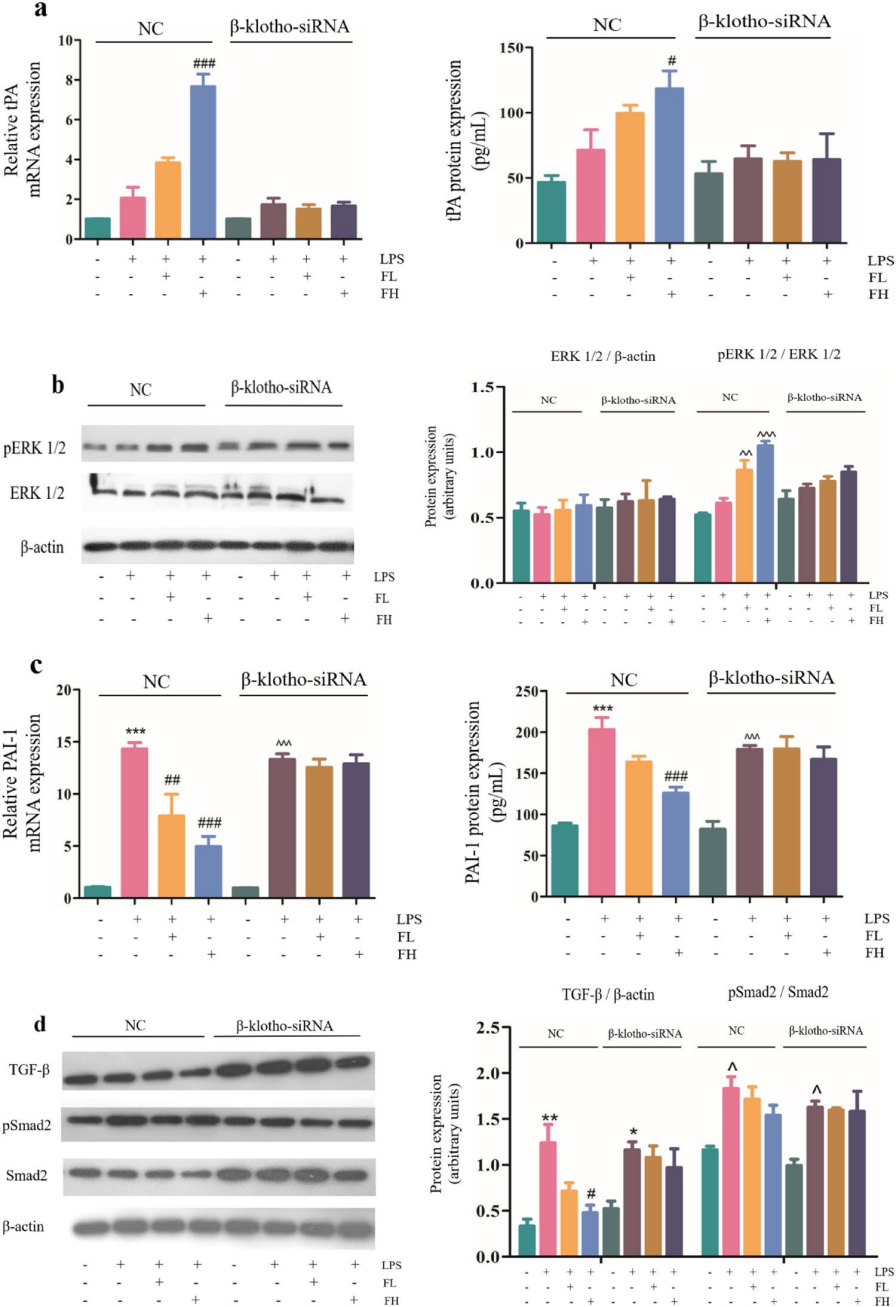
FGF-21 promotes tPA expression and inhibits PAI-1 expression in EA.hy926 cells. β-klotho-siRNA or NC siRNA was transiently transfected into EA.hy926 cells using Lipofectamine 3000 transfection reagent. Cells were treated with LPS (1000 μg/mL) with or without FGF-21 (FL: FGF-21 0.1 M, FH: FGF-21 1.0 M). tPA: tissue plasminogen activator; PAI-1: Plasminogen activator inhibitor 1; NC: nontransfection. (**a**) Analysis of tPA expression by real-time PCR and ELISA in EA.hy926 cells. (**b**) Western blot analysis of ERK1/2 phosphorylation levels in EA.hy926 cells. (**c**) Analysis of PAI-1 expression by real-time PCR and ELISA in EA.hy926 cells. (**d**) Western blot analysis of Smad2 phosphorylation levels and TGF-β expression in EA.hy926 cells. For all bar graphs, data are expressed as the mean±SD (n = 3). One-way ANOVA was used for multiple group comparisons, followed by Student’s two-tailed* t* test. *p < 0.05, **p < 0.01, ***p < 0.001 compared with the control NC group; ^#^*P* < 0.05, ^##^*P* < 0.01, ^###^*P* < 0.001 compared with the model NC group; ^^^*P* < 0.05, ^^^^*P* < 0.01, ^^^^^*P* < 0.001 compared with the control β-klotho-siRNA group.

The above results revealed that FGF-21 promotes tPA expression and inhibits PAI-1 expression in vivo. Next, we examined whether FGF-21 has the same effect in EA.hy926 cells. As expected, changes in the expression of tPA were consistent with the results observed in mice. In the NC group (transfection of non-targeting siRNA), expression of tPA was increased in LPS-treated cells compared with vehicle-treated cells, treatment with FGF-21 increased the expression of tPA in a dose-dependent manner, and levels of tPA were significantly increased in the high-dose FGF-21 group (^#^*P* < 0.05, ^###^* P* < 0.001). However, the results showed that expression of tPA in β-klotho knockdown cells treated with FGF-21 exhibited little change compared with LPS-treated cells (Fig. 6a). Similarly, when β-klotho was present, levels of phospho-ERK1/2 were increased in LPS-treated cells compared with vehicle-treated cells, and treatment with FGF-21 significantly increased levels of phospho-ERK1/2 in a dose-dependent manner (^^^^*P* < 0.01*, *^^^^^*P* < 0.001). However, western blot assays revealed that levels of phospho-ERK1/2 in β-klotho knockdown cells treated with FGF-21 displayed little change compared with LPS-treated cells (Fig. 6b). In addition, we found that phosphorylation levels of PAI-1, TGF-β and Smad2 were downregulated in FGF-21 treated cells compared with LPS-treated cells in the NC groupAfter β-klotho was inhibited, phosphorylation levels of PAI-1, TGF-β and Smad2 were not significantly reduced (Fig. 6c-d). These results indicate that FGF-21 promotes the expression of tPA and inhibits the expression of PAI by regulating the ERK1/2 and TGF-β/Smad2 pathways, respectively, in EA.hy926 cells.

## Discussion

FGF-21 has been shown to be a key endogenous regulator of numerous health benefits, including improving insulin sensitivity, and regulating inflammation, reducing adiposity, antioxidation and antiapoptosis^27–30^. Recent studies indicate that the development of CVD is associated with an increase in serum FGF-21 levels, which which are upregulated as a compensatory response to induce vessel protection^31,32^. Although the above studies affirm the preventive effect of FGF-21 against CVD, the role of FGF-21 in thrombosis is unknown. Thrombosis is caused by dysfunction of the normal hemostasis, anticoagulation and fibrinolysis systems. FVII is a vitamin K-dependent zymogen that is cleaved to the activated serpin protease FVIIa during hemostatic activation. FVIIa, as part of the total FVII in plasma, requires tissue factor (TF) for its proteolytic activity^33^. FVIIa-TF initiates the extrinsic coagulation pathway by activating coagulation factors IX and X, leading to thrombin generation and fibrin clot formation^34^. In vivo experiments, we demonstrated for the first time that FGF-21 decreases thrombus area, volume and burden by inhibiting FVII expression. Interestingly, we found that thrombus area, volume and burden in the FGF-21 treatment group were lower than those in the model group as assessed by OCT, but the difference was not significant. However, thrombus area, volume and burden in the FGF-21 prevention group were significantly lower than those in the model group. These results indicate that the prevention effect of FGF21 on thrombosis was better than the treatment effect. This phenomenon may be related to the systemic regulation of FGF21. Unlike enzymes, FGF21 does not act rapidly. As a regulator, it takes a certain amount of time to systematically regulate homeostasis. In addition, we found that FGF-21 inhibits platelet activation in mice with thrombi. Our findings demonstrate that FGF-21 displays an anticoagulation effect in vivo.

The plasminogen activator system is composed of two plasminogen activators (PAs, tissue-type tPA and the urokinase-type uPA), a specific uPA receptor (uPAR) and two PA inhibitors, PAI-1 and PAI-2, belonging to the serine protease inhibitor superfamily^35^. This system plays an important role in regulating thrombolysis^36,37^. Evidence suggests that the expression of tPA is observed on vascular endothelial cells in close contact with fibrin clots and is induced in pathological situations caused by or prone to thrombosis, such as ischemia, stroke and wounding^38–40^. tPA an FDA-approved drug for thrombus treatment, but the efficacy and safety of its therapeutic application are limited by narrow treatment time windows and side effects such as brain edema and hemorrhagic complications^41^. Using in vivo thrombosis studies, we demonstrated that administration of FGF-21 increased tPA expression and activity in thrombotic mice, suggesting that FGF-21 participates in the plasminogen activator system. D-dimer is a marker for evaluating fibrinolysis, which is a unique degradation product produced by plasmin-mediated proteolysis of cross-linked fibrin. In our study, administration of FGF-21 potently increased D-dimer generation in thrombotic mice. Next, we explored which signaling pathway is involved in regulating the expression of tPA induced by FGF-21. A large number of studies suggest that ERK1/2 plays an important role in tPA expression. Consistent with this, in vivo experiments revealed that levels of phospho-ERK1/2 was upregulated in response to FGF-21 treatment. To confirm these results, EA.hy926 cells were stimulated with LPS and then stimulated with FGF-21 at different concentrations. We observed that levels of phospho-ERK1/2 were increased in FGF-21 treated cells. We further explored whether this phenomenon was caused by FGF-21. β-klotho is widely believed to be a coreceptor required for FGF-21 binding to FGFRs, which are involved in signal transduction^42–46^. β-klotho was knocked down in EA.hy926 cells by transfection of β-klotho-siRNA (Supplementary Fig. 4). We detected few changes in the expression of phospho-ERK1/2 and tPA in FGF-21 treated cells, indicating that FGF-21 is involved in ERK1/2-mediated tPA expression.

tPA enzymatic activity can be inhibited by many factors^45^. PAI-1 inhibits plasminogen activation and fibrin degradation, and elevated PAI-1 levels in the plasma lead to thrombosis^47,48^. Administration of FGF-21 decreased PAI-1 expression in thrombotic mice. Several recent studies have suggested that the PAI-1 gene is highly induced by TGF-β in human cells. The addition of TGF-β to cultured human HepG2 hepatoma cells dramatically induced PAI gene expression. Coexpression of Smad2 or Smad3 with Smad4 also increases the expression of reporter genes driven by the PAI-1 promoter, and mutation of Smad4 leads to loss of the TGF-β response. Therefore, Smad proteins are involved in TGF-β-induced transcription of the PAI-1 gene. These findings are consistent with our results showing that treatment with FGF-21 inhibited the expression of TGF-β and levels of phospho-Smad2 in vivo and in vitro*.* Expression levels of TGF-β and phospho-Smad2 had no effects on FGF-21 treated cells when β-klotho was knocked down. Therefore, we concluded that FGF-21 inhibits PAI-1 expression by inhibiting the TGF-β/Smad2 pathway.

Recent studies have shown that inflammatory and proinflammatory cytokines induce a hypercoagulable state^22^. Coagulation disorders greatly affect the occurrence of acute thrombosis. The role of inflammatory cytokines in coagulation disorders has been illustrated by various studies^49^. Studies have shown that inflammatory factors activate the coagulation system and eventually lead to fibrin deposition^50^.The interaction between inflammation, coagulation, and fibrinolysis has been recently reviewed by Levi and colleagues^50^. As key factors in inflammatory reactions, mediators of inflammation are mutually amplified during thrombosis^51,52^. The anti-inflammatory effect of FGF-21 has been recognized. Consistent with these studies, we found that expression of CRP and IL-6 was increased in thrombotic mice, whereas treatment with FGF-21 blocked this increase and decreased thrombus area, volume and burden. In addition, we found that the levels of phospho-IκBα and nuclear p65 were increased in thrombotic mice and that FGF-21 treatment inhibited this increase. In addition, we observed similar results in our in vitro experiments. We found that FGF-21 administration did not mediate the levels of phospho-IκBα and nuclear p65 in β-klotho knockdown cells, further supporting that FGF-21 mediates the coagulation state by inhibiting the NF-κB pathway, subsequently ameliorating the overall inflammatory state.

At present, the risk of thrombolytic agents includes bleeding, which limits their clinical application. Identifying an ideal drug for prophylaxis and treatment of thrombotic disease without the risk of hemorrhage remains an unmet medical need. Notably, although FGF-21 has both anticoagulant and thrombolytic effects, it did not cause a notable risk of bleeding. These data suggest that FGF-21 is a safe and effective factor for regulating thrombosis and thrombolysis.

## Supplementary Information

Supplementary Information.
