# Supplementary material for: Fibroblast growth factor-21 as a novel metabolic factor for regulating thrombotic homeostasis
Source: Sci Rep. 2022 Jan 10;12:400. doi: 10.1038/s41598-021-00906-2 (PMC8748457; doi:10.1038/s41598-021-00906-2)
Supplement: Supplementary file 1 — Supplementary Information. [file 41598_2021_906_MOESM1_ESM.docx]

**Fibroblast growth factor-21 as a novel metabolic factor for** **regulating thrombotic homeostasis**

Shuai Li^b^, Haibo Jia^d^, Zhihang Liu^c^, Nan Wang^c^, Xiaochen Guo^c^, Muhua Cao^d^, Fang Fang^e^, Jiarui Yang^c^, Junyan Li^c^, Qi He^c^, Rui Guo^c^, Teng Zhang^c^, Kai Kang^c^, Zongbao Wang^c^, Shijie Liu^c^, Yukai Cao^c^, Xinghao Jiang^c^, Guiping Ren^c^, Kai Wang^e,^*, Bo Yu^d,^*, Wei Xiao^a,^*, Deshan Li^a, c ,^*.

a State Key Laboratory of New-tech for Chinese Medicine Pharmaceutical Process, Jiangsu Kanion Parmaceutical CO. LTD, Lianyungang, 222001, China.

b College of Life Sciences and Agriculture and Forestry, Qiqihar University, Qiqihar 161006, China

c Bio-pharmaceutical Lab, Life Science College, Northeast Agricultural University, Harbin, 150030, PR China.

d Department of Cardiology, The Key Laboratory of Myocardial Ischemia, Chinese Ministry of Education, The 2nd Affiliated Hospital of Harbin Medical University, Harbin, 150030, PR China.

e Molecular Imaging Research Center, Harbin Medical University, TOF-PET/CT/MR center, The Fourth Hospital of Harbin Medical University, Harbin, 150030, PR China.

*Correspondence: Jiangsu Kanion Parmaceutical CO. LTD, Jiangsu, Lianyungang, 222001; State Key Laboratory of New-tech for Chinese Medicine Pharmaceutical Process，Jiangsu Lianyungang 222001, China. E-mail: deshanli@163.com

*Correspondence: Jiangsu Kanion Parmaceutical CO. LTD, Jiangsu, Lianyungang, 222001; State Key Laboratory of New-tech for Chinese Medicine Pharmaceutical Process，Jiangsu Lianyungang 222001, China. E-mail: xw_kanion@163.com

*Correspondence: Department of Cardiology, The 2nd Affiliated Hospital of Harbin Medical University, The Key Laboratory of Myocardial Ischemia, Chinese Ministry of Education, 246 Xuefu Road, Nangang District, Harbin, Heilongjiang 150086, P.R. China. E-mail: yubodr@163.com

*Correspondence: Molecular Imaging Research Center, Harbin Medical University, TOF-PET/CT/MR center, The Fourth Hospital of Harbin Medical University, Harbin, Heilongjiang, China. E-mail: wangkai@hrbmu.edu.cn

| Author | E-mail | Phone |
| --- | --- | --- |
| Shuai Li^b^ | ls90729@163.com | 15204528699 |
| Haibo Jia^d^ | 616662721@qq.com | 18846072376 |
| Zhihang Liu^c^ | 1269674345@qq.com; | 15663865937 |
| Nan Wang^c^ | 848661124@qq.com | 15069880869 |
| Xiaochen Guo^c^ | 676553202@qq.com | 13204662306 |
| Muhua Cao^d^ | 1159490506@qq.com | 18846072543 |
| Fang Fang^e^ | 1005421953@qq.com | 18846072396 |
| Jiarui Yang^c^ | 1260481892@qq.com | 18846441952 |
| Junyan Li^c^ | 2545086445@qq.com | 13936599263 |
| Qi He^c^ | 499621167@qq.com | 18846177959 |
| Rui Guo | 1820179785@qq.com | 15204606967 |
| Teng Zhang^c^ | 1021127392@qq.com | 18846087308 |
| Kai Kang^c^ | 2715043737@qq.com | 15546095197 |
| Zongbao Wang^c^ | 1052695316@qq.com | 18846079864 |
| Shijie Liu^c^ | 3075027285@qq.com | 18846072533 |
| Yukai Cao^c^ | 117977894@qq.com | 18846072411 |
| Xinghao Jiang^c^ | 1101833649@qq.com | 18846441952 |
| Guiping Ren^c^ | 516702737@qq.com | 15945183992 |
| Kai Wang^e,^* | wangkai@hrbmu.edu.cn | 13796601005 |
| Bo Yu^c,^* | 965422155@qq.com | 13804585601 |
| Wei Xiao^c,^* | xw_kanion@163.com | 18905171327 |
| Deshan Li^a,b,^* | deshanli@163.com | 18886775632 |

**Running title:** FGF-21 regulates thrombotic homeostasis

**Funding:** This work was supported by National Key R&D Program of China (2017YFD0501102, 2016YFD0501003)，Heilongjiang Province Fundamental Research Funds for Young Scholar (135409219).


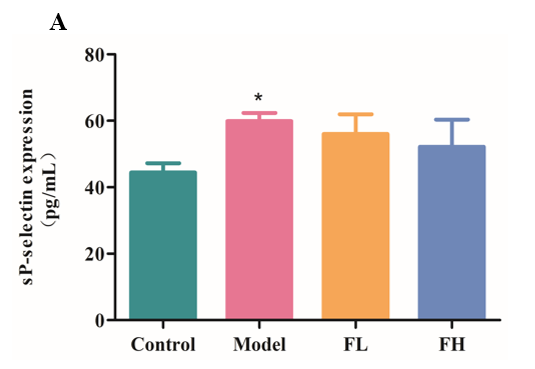


Supplemental Figure 1. ELISA of sP-selectin expression in the serum. Mice were administered Ca (3 mg/kg) via intraperitoneal injection and LPS (50 μg/kg) via the tail vein 16 h later. Then, FGF-21 was administered to the mice once daily for 5 days. C: normal control; M: Ca-LPS-induced thrombosis mice; FL: thrombosis mice treated with low dose FGF-21 (5 mg/kg); FH: thrombosis mice treated with high dose FGF-21 (10 mg/kg). For all bar graphs, data are expressed as the mean±SD (n=6). One-way ANOVA was used for multiple group comparisons, followed by Student’s two-tailed t-test. **p<0.05* compared with the control.


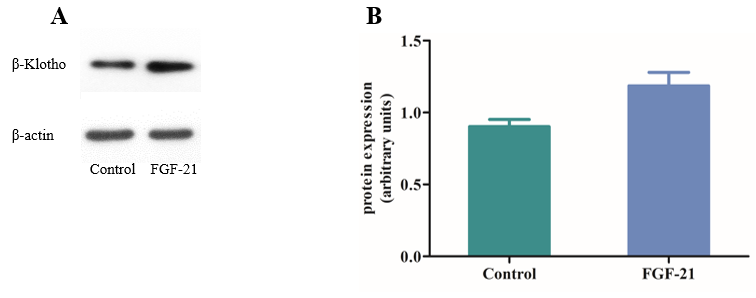


Supplemental Figure 2. Detection of β-klotho in EA.hy926 cells. Cells were treated with or without FGF-21.


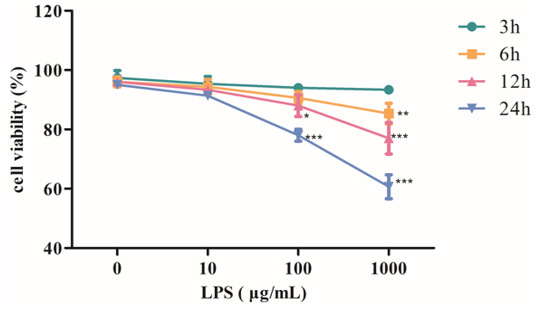


Supplemental Figure 3. Influence of different concentration LPS on cell growth. EA.hy926 cells were treated with different concentrations of LPS (10 μg/mL, 100 μg/mL and 1000 μg/mL) for different time (3 h, 6 h, 12 h, 24 h) and detected by MTT. For all bar graphs, data are expressed as the mean±SD (n=3). One-way ANOVA was used for multiple group comparisons, followed by Student’s two-tailed t-test. *^*^P<0.05, ^**^P<0.01, ^***^P<0.001* compared with normal control.


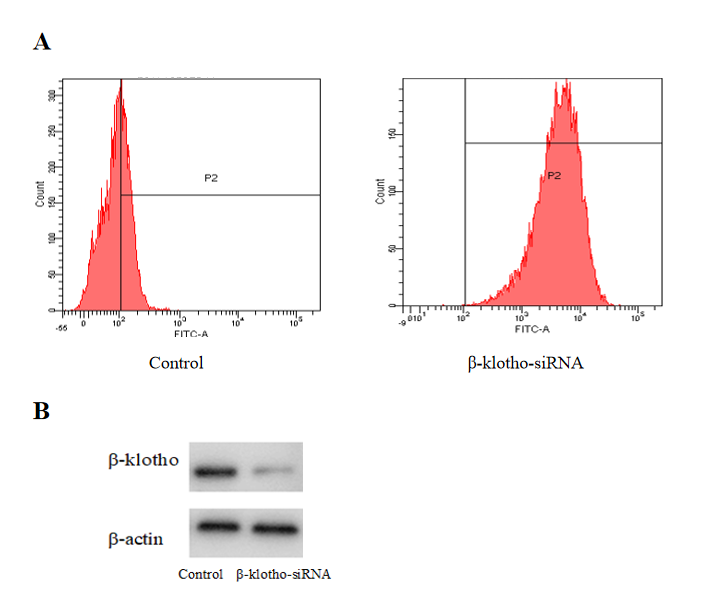


Supplemental Figure 4. β-klotho knock down in EA.hy926 cells by transfection of β-klotho-siRNA. (A) The transfection efficiency of β-klotho-siRNA was detected by flow cytometry. (B) Analysis of β-klotho knockdown by Western blot.

The original image of the western blot


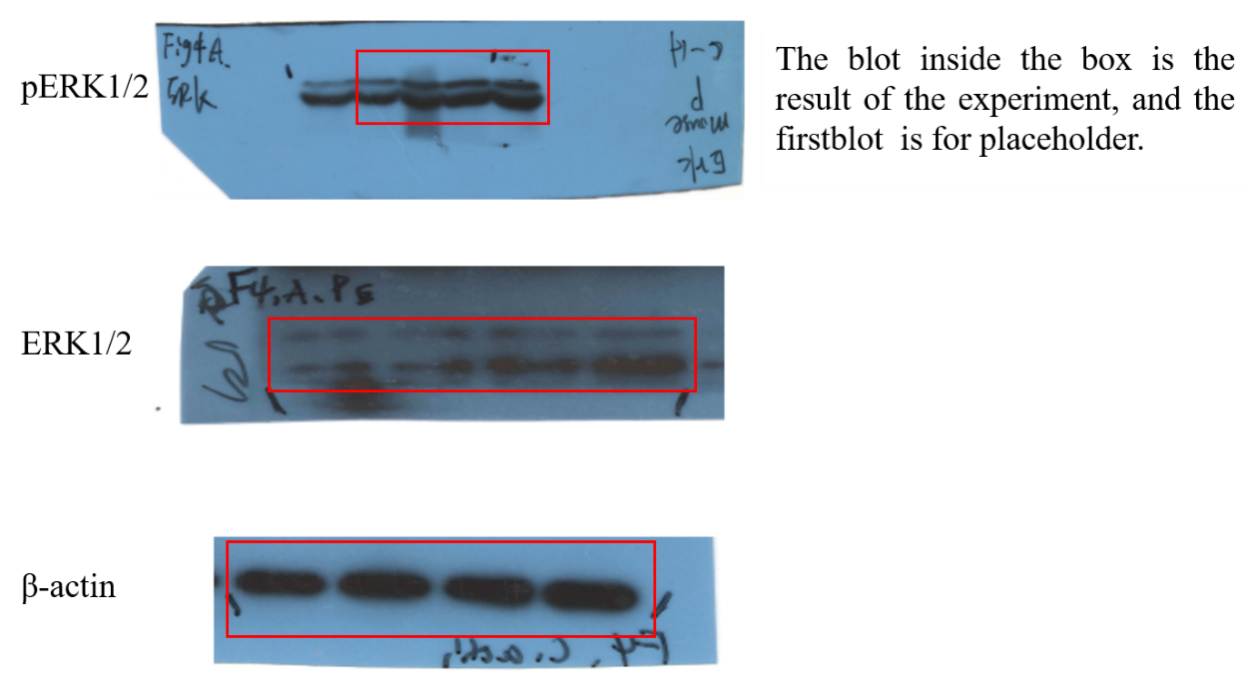


The blot inside the box is the result of the experiment, and the first-blot is for placeholder.

ERK1/2

pERK1/2

β-actin

The original image of Figure 3d. Western blot analysis of ERK1/2 phosphorylation level in mice. The blots inside the red box are the results in the Figure 3d, and from left to right are the Control, Model, FL and FH groups.


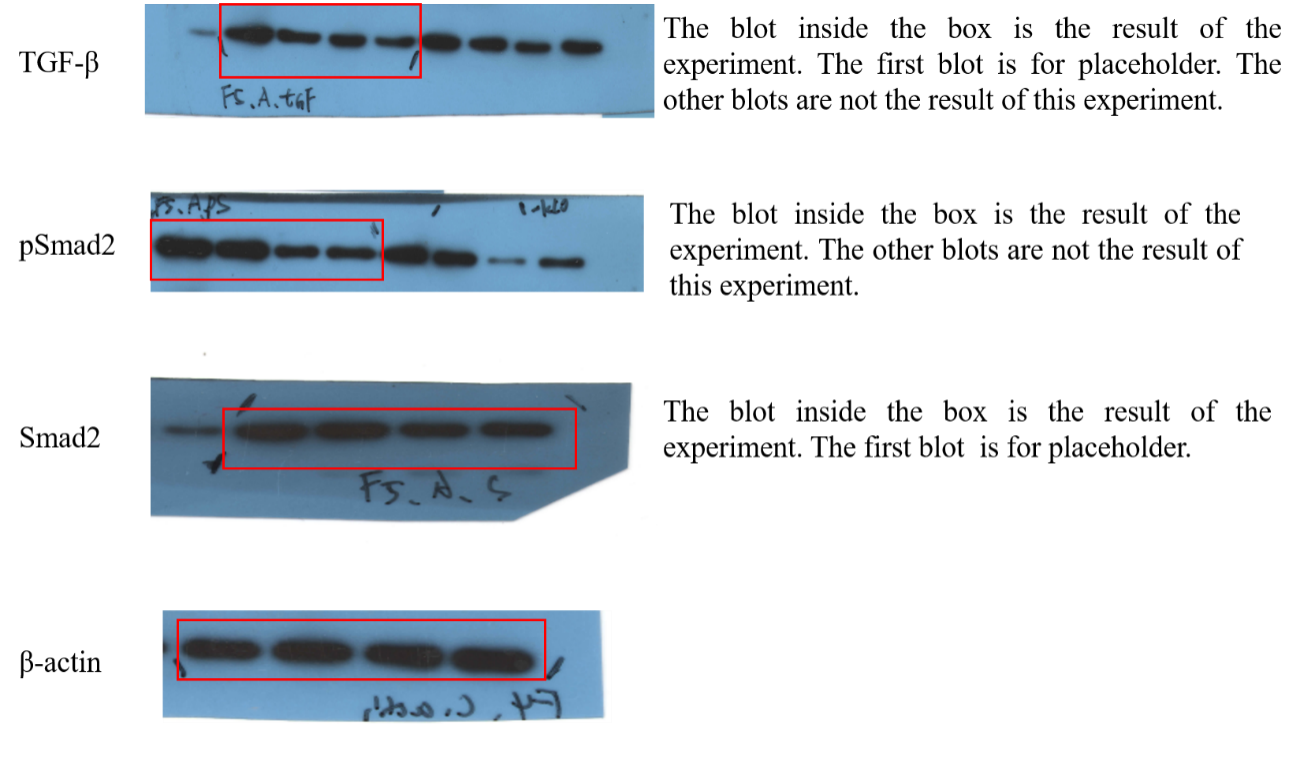


The blot inside the box is the result of the experiment. The other blots are not the result of this experiment.

The blot inside the box is the result of the experiment. The first-blot is for placeholder.

The blot inside the box is the result of the experiment. The first-blot is for placeholder. The other blots are not the result of this experiment.

The original image of Figure 3f. Western blot analysis of Smad2 phosphorylation level and TGF-β expression in mice. The blots inside the red box are the results in the Figure 3f, and from left to right are the Control, Model, FL and FH groups.


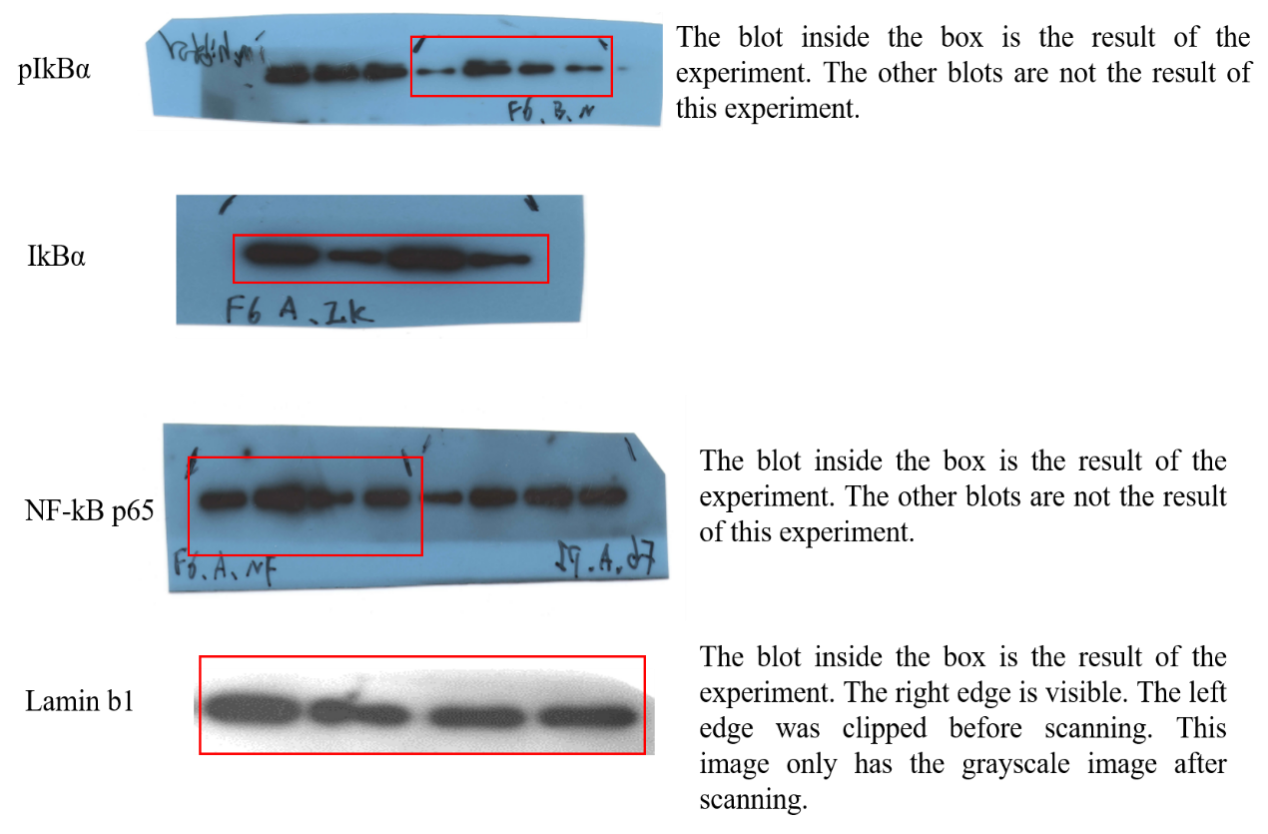


The blot inside the box is the result of the experiment. The right edge is visible. The left edge was clipped before scanning. This image only has the grayscale image after scanning.

The blot inside the box is the result of the experiment. The other blots are not the result of this experiment.

The blot inside the box is the result of the experiment. The other blots are not the result of this experiment.

The original image of Figure 4d. Western blot analysis of phospho-IκB and p65 expression in mice. The blots inside the red box are the results in the Figure 4d, and from left to right are the Control, Model, FL and FH groups.


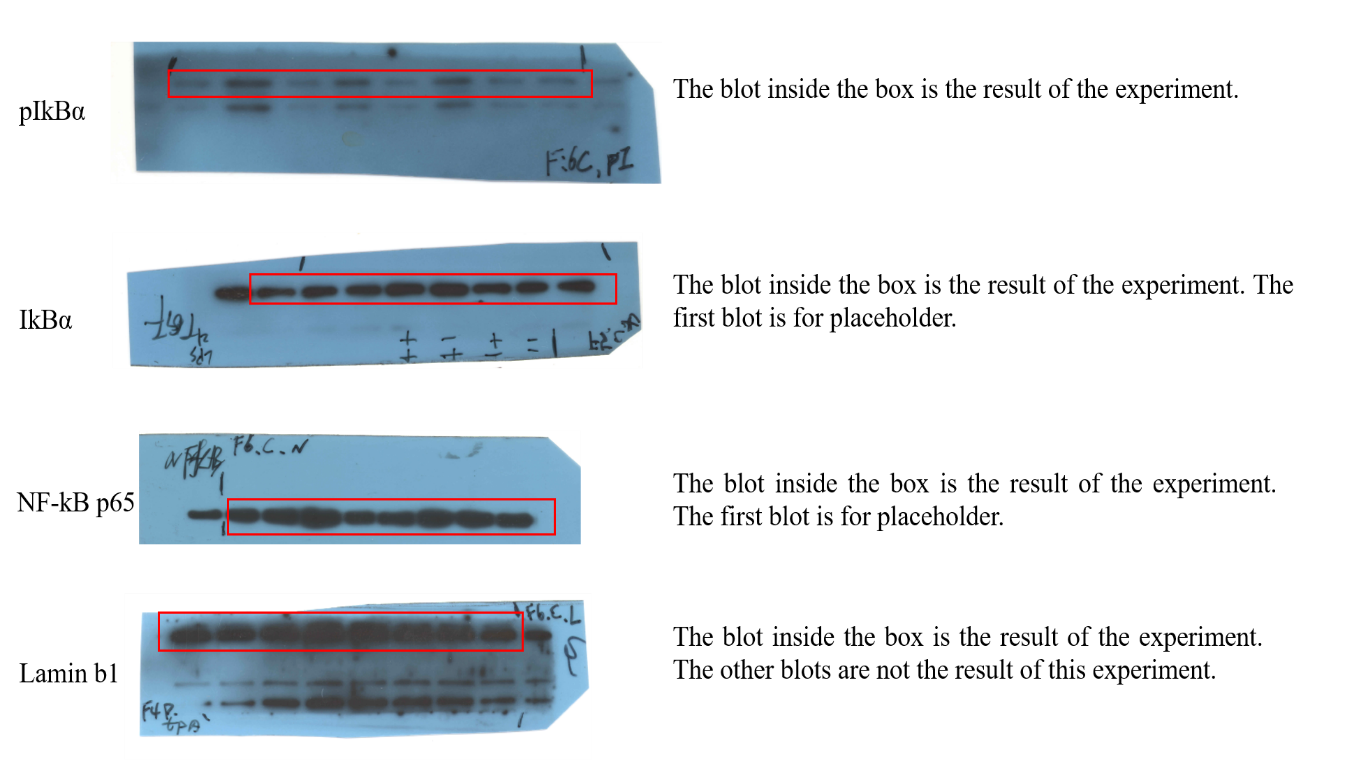


The blot inside the box is the result of the experiment. The other blots are not the result of this experiment.

The blot inside the box is the result of the experiment. The first-blot is for placeholder.

The blot inside the box is the result of the experiment. The first-blot is for placeholder.

The blot inside the box is the result of the experiment.

The original image of Figure 5d. Western blot analysis of phospho-IκB and p65 expression in EA.hy926 cells. The blots inside the red box are the results in the Figure 5d.


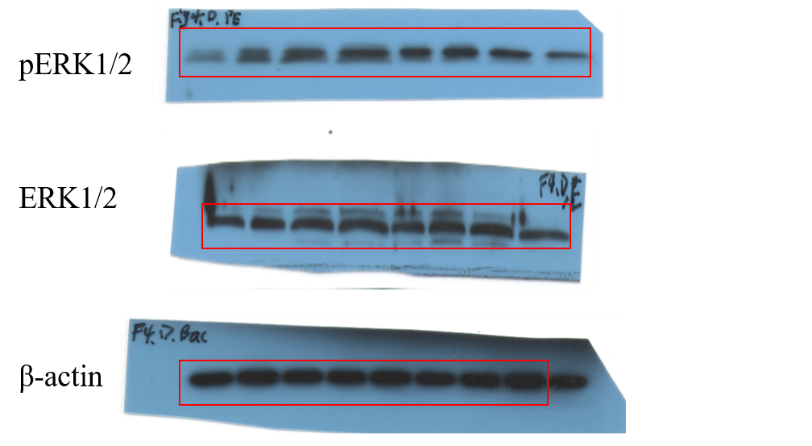


The blot inside the box is the result of the experiment. The last-blot is for placeholder.

The original image of Figure 6b. Western blot analysis of ERK1/2 phosphorylation level in EA.hy926 cells. The blots inside the red box are the results in the Figure 6b.


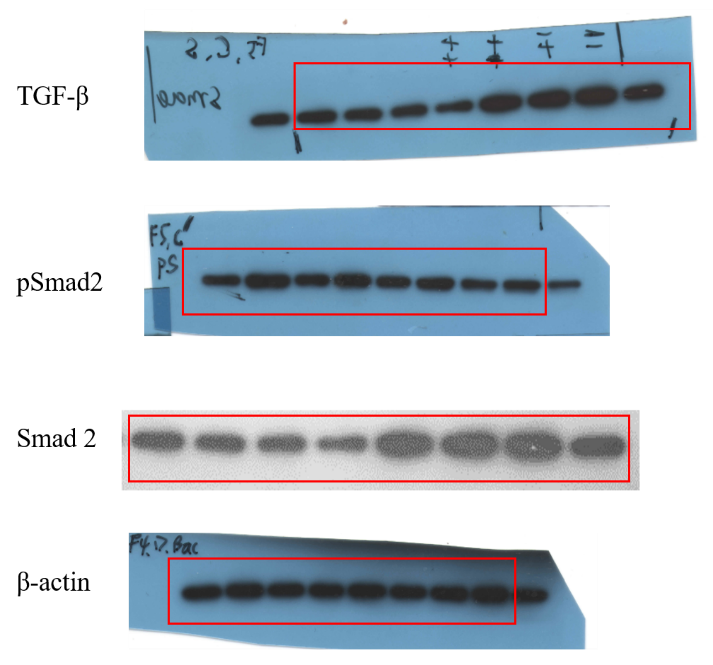


The blot inside the box is the result of the experiment. The last-blot is for placeholder.

The blot inside the box is the result of the experiment. The first-blot is for placeholder.

This image only has the grayscale image after scanning.

The blot inside the box is the result of the experiment. The last-blot is for placeholder.

The original image of Figure 6d. Western blot analysis of Smad2 phosphorylation level and TGF-β expression EA.hy926 cells. The blots inside the red box are the results in the Figure 6d.


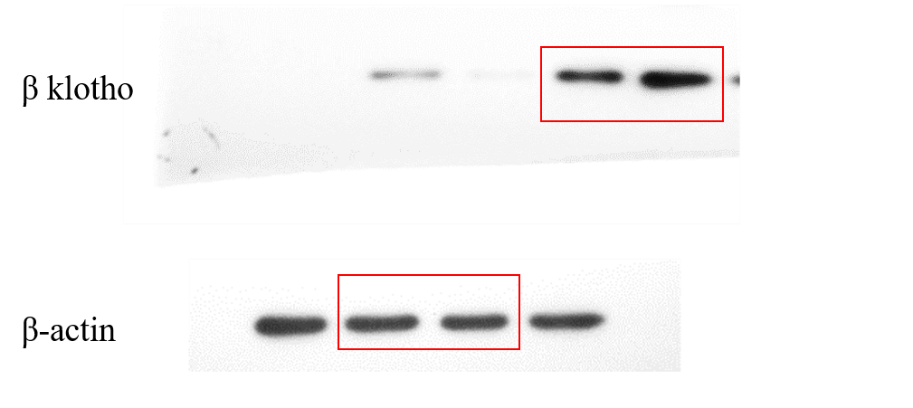


The blot inside the box is the result of the experiment. The last-blot is for placeholder. This image only has the grayscale image after scanning.

The blot inside the box is the result of the experiment. The other blots are not the result of this experiment. This image only has the grayscale image after scanning.

The original image of Supplemental Figure 2A. Detection of β-klotho in EA.hy926 cells. Cells were treated with or without FGF-21. The blots inside the red box are the results in the Supplemental Figure 2A.


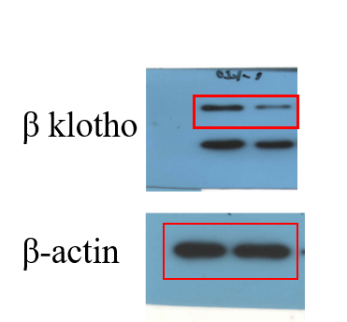


The left edge is visible. The right edge was clipped before scanning.

The blot inside the box is the result of the experiment. The other blots are not the result of this experiment. The left edge is visible. The right edge was clipped before scanning.

The original image of Supplemental Figure 4B. Analysis of β-klotho knockdown by Western blot in EA. hy 926 cells. The blots inside the red box are the results in the Supplemental Figure 4B.
